# Comparative evaluation of the physicochemical and biological properties of calcium silicate-based pulp capping materials

**DOI:** 10.3389/fdmed.2025.1737941

**Published:** 2025-12-12

**Authors:** Juan Carlos Hernández-Cabanillas, Louis Hardan, Carlos Enrique Cuevas-Suárez, Iván Olivares-Acosta, Anh Tuan Dang, Vincenzo Tosco, Naji Kharouf, Monika Lukomska-Szymanska, Youssef Haikel, Rim Bourgi

**Affiliations:** 1 Facultad de Ciencias de la Salud Unidad Valle de las Palmas—UABC, Blvd Universitario, Tijuana, Mexico; 2 Department of Restorative and Esthetic Dentistry, Faculty of Dental Medicine, Saint-Joseph University of Beirut, Beirut, Lebanon; 3 Dental Materials Laboratory, Academic Area of Dentistry, Autonomous University of Hidalgo State, Circuito Ex Hacienda La Concepción S/N, San Agustín Tlaxiaca, Hidalgo, Mexico; 4 Faculty of Dentistry, Haiphong University of Medicine and Pharmacy, Haiphong, Vietnam; 5 Department of Odonto-Stomatology, Haiphong Medical University Hospital, Haiphong, Vietnam; 6 Department of Clinical Sciences and Stomatology, Università Politecnica Delle Marche, Ancona, Italy; 7 Department of Biomaterials and Bioengineering, INSERM UMR_S 1121, University of Strasbourg, Strasbourg, France; 8 Department of Endodontics and Conservative Dentistry, Faculty of Dental Medicine, University of Strasbourg, Strasbourg, France; 9 Department of General Dentistry, Medical University of Lodz, Lodz, Poland; 10 Pôle de Médecine et Chirurgie Bucco-Dentaire, Hôpital Civil, Hôpitaux Universitaire de Strasbourg, Strasbourg, France; 11 Department of Restorative Sciences, Faculty of Dentistry, Beirut Arab University, Beirut, Lebanon

**Keywords:** biocompatibility, biodentine, biomineralization, calcium silicate, compressive strength, MTA, pulp capping, TheraCal

## Abstract

**Background:**

Understanding the performance of calcium silicate-based pulp capping materials is essential for clinicians seeking to preserve pulp vitality in cases of carious exposure, dental trauma, or developmental defects.

**Objective:**

This study aimed to compare the physicochemical and biological properties of four pulp capping materials: Mineral Trioxide Aggregate (MTA), Biodentine®, TheraCal LC, and TheraCal PT.

**Methods:**

Compressive strength, shear bond strength to composite resin, radiopacity, *in vitro* biomineralization, and cytocompatibility with human dental pulp stem cells (hDPSCs) were evaluated under standardized conditions. Statistical analysis was performed using one-way analysis of variance (ANOVA) and Tukey's *post hoc* test (*α* = 0.05).

**Results:**

TheraCal PT exhibited the highest compressive and bond strength (*p* < 0.001), while MTA showed the greatest radiopacity (*p* < 0.001). Biodentine and MTA demonstrated superior biomineralization with abundant calcium phosphate crystal formation. In cell viability assays, Biodentine and TheraCal PT performed similarly to the control (*p* > 0.024), whereas Biodentine and TheraCal LC showed significantly reduced viability (*p* < 0.001).

**Conclusions:**

Resin-modified materials offer advantages in mechanical performance and bonding but may compromise biomineralization and biocompatibility. Water-based materials like MTA remain superior in bioactivity and cellular response. Clinicians should balance physical properties with biological outcomes when selecting pulp capping agents.

## 1 Introduction

Dental caries is a prevalent condition that affects over 93% of adults aged 20 years and older (1). When untreated, caries can lead to pulp infection or necrosis (2). Historically, treatment involved the non-selective removal of all carious dentin. However, current strategies have shifted toward minimally invasive approaches aimed at preserving pulp vitality (3). One such technique is direct pulp capping, which involves applying a bioactive material to the exposed pulp. These materials release calcium, phosphate, and fluoride ions that chemically interact with dental tissues, leading to the formation of a dentin-pulp complex and promoting tissue repair (4).

Since the development of calcium silicate-based materials in the late 20th century, several compounds have been evaluated for their potential in pulp capping procedures (5). These materials are designed to maintain pulp vitality and stimulate dentin bridge formation (6). *In vitro* experimental studies have played a pivotal role in characterizing the physical, mechanical, and biological properties of these materials to predict their clinical performance (7–9). Among the most widely studied materials are Biodentine® (Septodont), Mineral Trioxide Aggregate (MTA), TheraCal Light-Cured (LC), and TheraCal Pulp Therapy (PT) (both from Bisco company) (10).

Calcium silicate-based materials have demonstrated favorable properties, such as high pH, antimicrobial activity, and the ability to release calcium ions that support hard tissue formation (11). Nonetheless, newer resin-modified versions like TheraCal LC and PT have emerged, offering easier handling and faster setting times (12). Despite these advantages, concerns remain regarding their cytotoxicity and long-term bioactivity (10). This has raised the need for more comprehensive laboratory studies to better understand their mechanical strength, bond performance with resin composites, radiopacity, biomineralization capacity, and cellular compatibility.

Understanding the performance of these materials is crucial for clinicians aiming to preserve pulp vitality in cases of carious exposure, trauma, or developmental defects (13). While traditional materials such as MTA and Biodentine have demonstrated consistent clinical outcomes (14, 15), the introduction of resin-based materials requires critical assessment to ensure their safe and effective use (16). This research aims to contribute valuable data that can support evidence-based decision-making in restorative and endodontic procedures.

The primary goal of this study was to characterize and compare the physicochemical and biological properties of four commonly used direct pulp capping agents: MTA, Biodentine, TheraCal LC, and TheraCal PT. These materials were selected due to their widespread clinical use and varying compositions—ranging from traditional bioceramics to resin-modified calcium silicates. A thorough evaluation of their compressive strength, bonding resistance, radiopacity, *in vitro* biomineralization, and cytocompatibility was conducted to determine their suitability for long-term clinical application. The null hypothesis of this study is that resin-modified calcium silicate-based materials (TheraCal LC and PT) will exhibit similar mechanical, chemical, and biological properties compared to conventional water-based bioceramic materials (MTA and Biodentine).

## 2 Materials and methods

Four commercially available direct pulp capping materials were evaluated in this study: Biodentine® (Septodont, Saint-Maur-des-Fossés, France), mineral trioxide aggregate (MTA; Viarden, Mexico), TheraCal LC, and TheraCal PT (Bisco Inc., Schaumburg, IL, USA). All specimens were prepared under standardized laboratory conditions using materials from the same batch number for consistency. Specimens with visible surface defects or irregular dimensions were excluded from the analysis. Sample size calculation was performed separately for each dependent variable, considering a power of 0.8 and an *α* = 0.05. The minimum detectable difference in means and the standard deviation used for the calculation were obtained from previous studies.

### 2.1 Compressive strength test

Five cylindrical specimens (6 mm diameter × 4 mm height) were prepared for each material. All materials were mixed according to the manufacturers' instructions and then filled into a separable cylindrical stainless-steel mold of 4 mm in diameter and 6 mm in height. For water-based cements, after filling the mixture, both sides of the mold were covered by glass plates, then kept at 37 °C and 100% humidity for 24 h. On the other hand, resin-based materials were polymerized for 20 s per side using a 1,000 mW/cm² HeptaLux curing light (Xpedent, Guilin, China).

After completely setting of the materials, each specimen was carefully removed from the mold and its new dimensions (diameter and height) were measured using micrometer (Mitutoyo, Kanagawa, Japan). The compressive strength was measured using a universal testing machine (Instron 4,465, Norwood, MA, USA) with a 1 kN load cell at a crosshead speed of 1 mm/min. Compressive strength (MPa) was calculated using theformula: CS = 4F/πd², where F is the maximum load and *d* is the specimen diameter.

### 2.2 Shear bond strength to resin composite materials

Five acrylic blocks with central cylindrical cavities (5 mm diameter × 2 mm depth) were filled with the testing materials. Light-cured materials were polymerized for 20 s at 1,000 mW/cm², while water-based cements were stored at 37 °C under 100% humidity for 24 h. After setting, each surface was etched with 37% phosphoric acid for 30 s, rinsed, and dried. Then, Single Bond Universal adhesive (3M ESPE, St. Paul, MN, USA) was applied and light-cured. A silicone matrix with two cylindrical holes (1.4 mm diameter) was filled with Filtek Z250 resin composite (3M ESPE, St. Paul, MN, USA) and light-cured for 20 s. The matrix was removed, and samples were stored in distilled water at 37 °C for 24 h before testing. Shear bond strength was measured using a universal testing machine (Instron 1,145, Norwood, MA, USA) at a crosshead speed of 1.0 mm/min. Results were reported in MPa.

### 2.3 Radiopacity test

Three cylindrical specimens (6 mm diameter × 1 mm thickness) of each material were prepared. Digital radiographs were obtained using a phosphor plate system (VistaScan; Dürr Dental GmbH, Germany) with an aluminium step wedge (0.5–5.0 mm thickness, in 0.5 mm increments) under standard conditions (70 kV, 8 mA, 0.4 s exposure time, 400 mm film-focus distance). Images were saved in TIFF format and analyzed using Photoshop software (Adobe Systems Inc., San José, CA, USA). Radiopacity was determined by comparing the grayscale pixel density of the samples with the step wedge and expressed in millimetres of aluminium (mm Al).

### 2.4 *In vitro* biomineralization assessment

Simulated body fluid solution (SBF) was prepared according to Kokubo's methodology (17). Disc samples (5 mm diameter × 2 mm height) were made for each group (*n* = 6); the samples were prepared as explained before. The specimens were then immersed vertically in the SBF for 21 days at 37 °C (Felisa Stove). The SBF solution was changed weekly. After 21 days the cement discs were gold sputtered for superficial Scanning Electron Microscopy (SEM) images (JEOL JSM5600-LV).

### 2.5 Cell viability assay

The cytotoxicity test was performed according to International Organization for Standardization (ISO) specification 10,993–5:2009 (17). The specimens (*n* = 3) were placed in 24-well plates containing DMEM (Dulbecco's Modified Eagle's medium) and stored at 37 °C with a pH of 7.2 for 24 h. The volume of DMEM used for each sample preparation protocol was calculated in accordance with the ISO 10993–12 standard. During the 24 h incubation period, the conditioned medium was expected.

Human dental pulp stem cells were cultured in modified eagle's minimal essential medium (MEM, Sigma-Aldrich, St Luis, MO, USA) supplemented with 10% fetal bovine serum (FBS, Sigma-Aldrich), 10,000 IU/mL penicillin, 100 mg/mL streptomycin (Penstrep, Sigma-Aldrich), and 1% Glutamax (Life Technologies, Gibco, Grand Island, NE, USA). Cells were incubated at 37 °C in a humidified atmosphere of 5% CO_2_.

Cells (2 × 105 cells/mL) were inoculated into a 96-microwell plate and incubated for 48 h to achieve complete adherence to the culture dish. After the incubation period, the culture medium was replaced with an equal volume of the conditioned medium containing the eluate from each specimen (200 μL per well). The plate was then incubated at 37 °C with 5% CO_2_ for an additional 24 h.

After the incubation period, the culture medium was replaced with MTT (0.2 mg/mL) dissolved in MEM medium, and the cells were incubated for 7 h at 37 °C, 95% humidity and 5% CO_2_. The formazan products were then dissolved with dimethyl sulfoxide (100 µL, DMSO, Karal, León de los Aldama, Mexico), and absorbance at 570 nm was determined using a microplate reader (Thermo Scientific, Waltham, MA, USA).

### 2.6 Statistical analysis

All data were evaluated for normality and homogeneity of variance using the Shapiro–Wilk and Levene tests, respectively. One-way analysis of variance (ANOVA) followed by Tukey's *post hoc* test was used for pairwise comparisons, with a significance level set at *α* = 0.05. Statistical analyses were performed using Jamovi software (version 2.5.6).

## 3 Results

**Figure 1:**
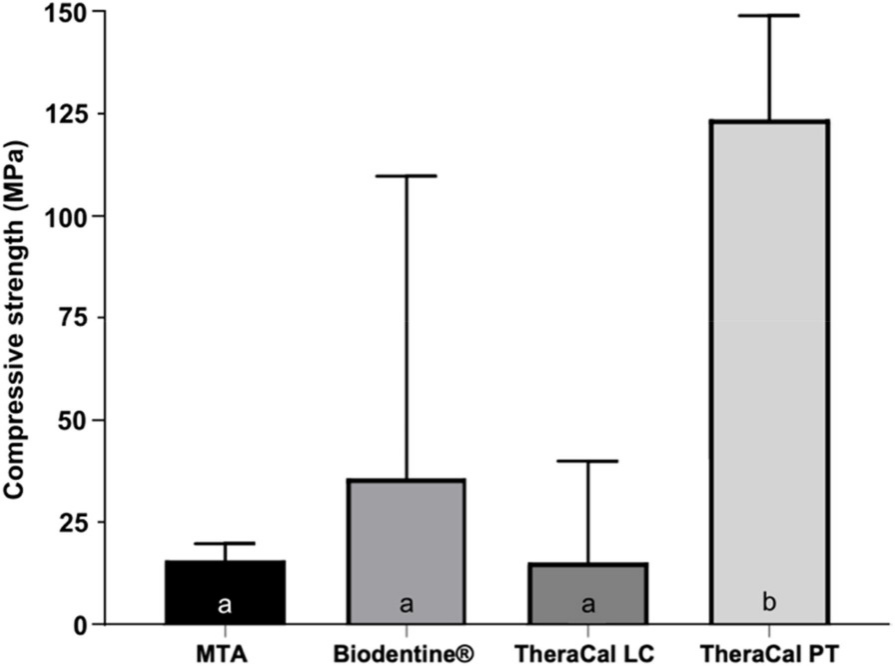
Compressive strength (MPa) of the materials tested. Different lowercase letters indicate the presence of statistically significant differences (*p* < 0.05).

Statistical analysis using one-way ANOVA revealed significant differences in compressive strength among the tested materials (Figure 1; *p* = 0.001). Tukey's *post hoc* test indicated that TheraCal PT exhibited the highest compressive strength, with statistically significant differences compared to all other groups (*p* < 0.001). No statistically significant differences were observed between MTA, Biodentine®, and TheraCal LC (*p* > 0.052).

**Figure 2:**
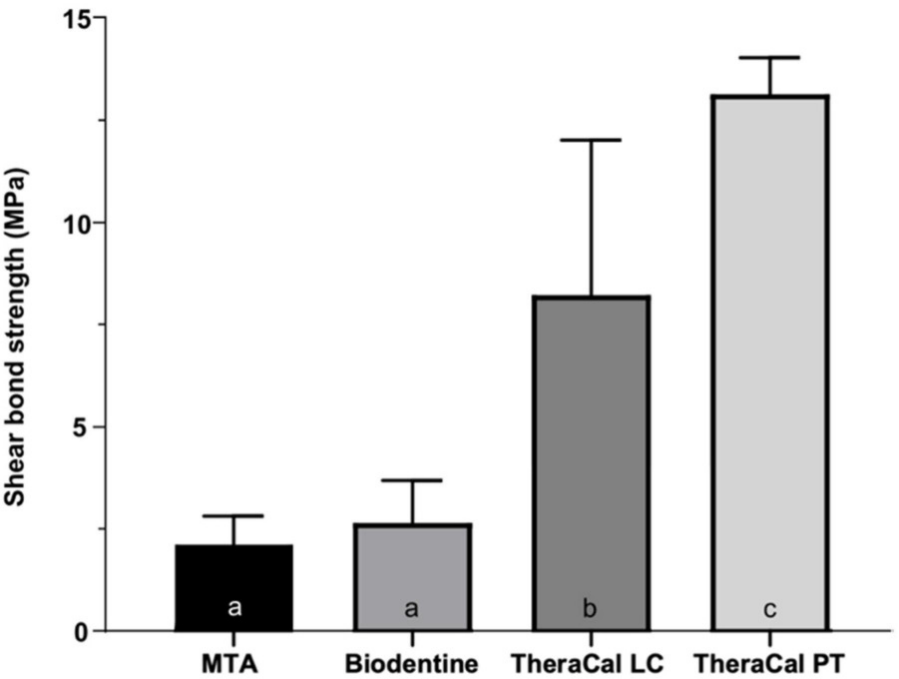
Shear bond strength (MPa) to resin composite. Different lowercase letters indicate the presence of statistically significant differences (*p* < 0.05).

Regarding bond strength to resin-based materials (Figure 2), one-way ANOVA showed statistically significant differences among the materials (*p* = 0.001). Tukey's *post hoc* test demonstrated that TheraCal PT presented the highest bond strength (*p* < 0.001). TheraCal LC also exhibited significantly higher values than MTA and Biodentine (*p* < 0.003). No statistically significant differences were observed between MTA and Biodentine (*p* = 0.976).

**Figure 3:**
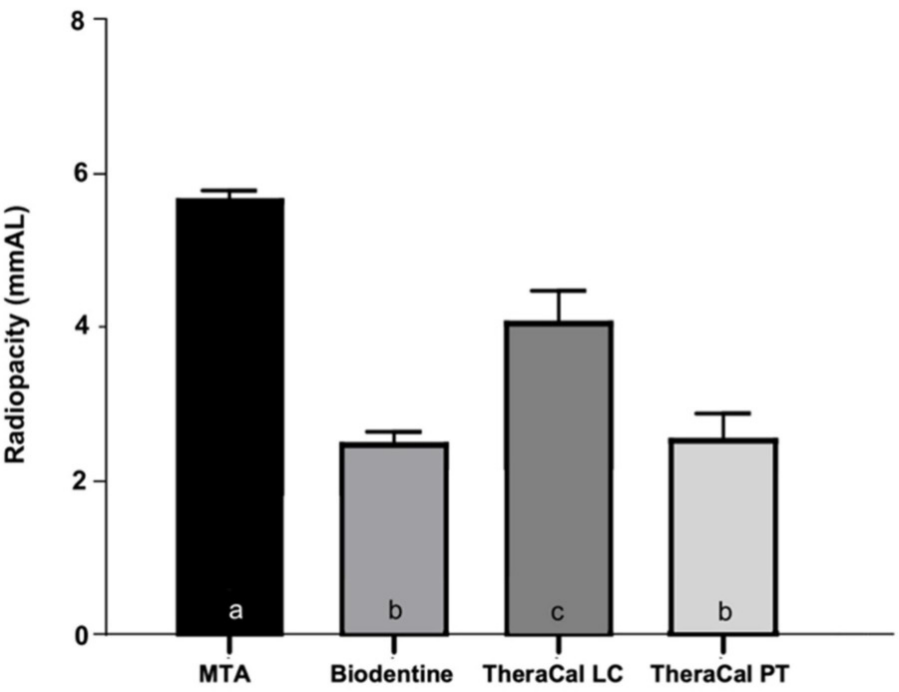
Radiopacity of the materials tested. Different lowercase letters indicate the presence of statistically significant differences (*p* < 0.05).

Figure 3 shows the radiopacity values of the materials tested. According to the statistical analysis, the radiopacity differed significantly among the groups (*p* = 0.001). MTA exhibited the highest radiopacity (*p* < 0.001), followed by TheraCal LC, which showed significantly higher values than both Biodentine and TheraCal PT (*p* < 0.002). No significant differences were observed between Biodentine and TheraCal PT (*p* = 0.994).

**Figure 4:**
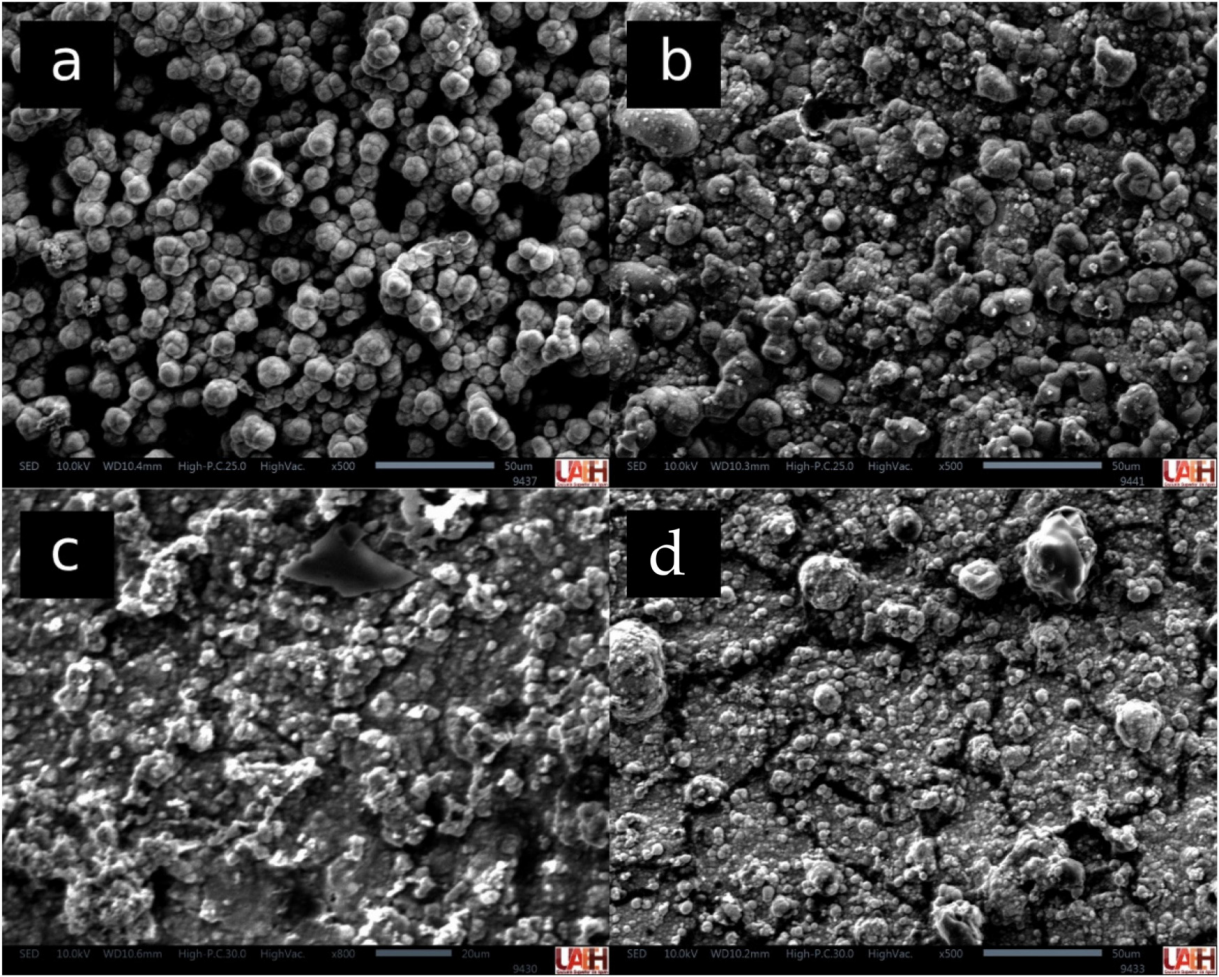
SEM images from the surface of biodentine **(a)**, MTA **(b)**, theraCal LC **(c)**, and theraCal PT **(d)**.

Figure 4 shows the SEM images of the surface of the materials after the *in vitro* biomineralization assay. SEM revealed distinct differences in surface mineralization. Biodentine exhibited the most pronounced formation of calcium phosphate crystals, primarily with spheroidal morphology. MTA also showed surface deposition of hydroxyapatite, characterized by spherulitic and spheroidal structures. In contrast, TheraCal LC and TheraCal PT demonstrated minimal calcium phosphate formation on their surfaces.

**Figure 5:**
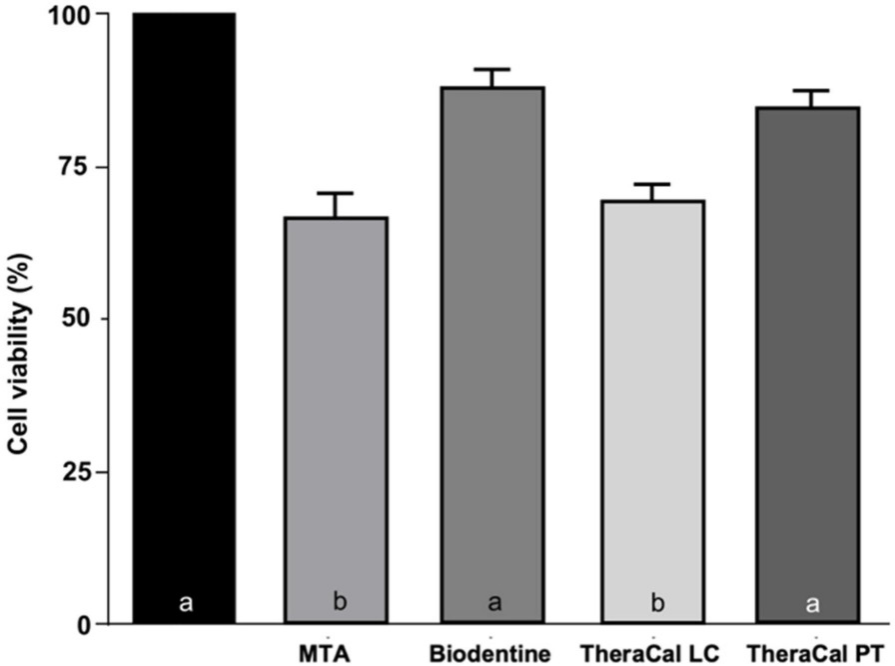
Cell viability of the materials tested. Different lowercase letters indicate the presence of statistically significant differences (*p* < 0.05).

Cell viability results are shown in Figure 5. Significant differences in cell viability under indirect contact with hDPSCs were observed among the tested materials (*p* = 0.001). Tukey's *post hoc* analysis showed no statistically significant differences between the control group (PBS), Biodentine, and TheraCal PT (*p* > 0.024). In contrast, MTA and TheraCal LC demonstrated significantly lower cell viability compared to the control group (*p* < 0.001).

## 4 Discussion

This study revealed significant differences in the physicochemical and biological properties among the four pulp capping materials evaluated, leading to the rejection of the null hypothesis. The key findings demonstrate a clear trade-off: resin-modified materials excel in mechanical properties but show limitations in bioactivity, whereas water-based bioceramics exhibit superior biological performance.

TheraCal PT demonstrated the highest compressive strength, which can be attributed to its dual-cure polymerization mechanism that ensures more complete monomer conversion and higher cross-link density compared to single-cure materials (17, 18). Its resin-based formulation containing Bis-GMA, UDMA, and PEGDMA contributes to enhanced mechanical stability (19). In contrast, the hydration-based setting reactions of MTA and Biodentine typically result in more porous matrices with lower early-stage strength (21, 22), as confirmed by our results.

A crucial finding emerged when comparing compressive strength with shear bond strength, particularly for TheraCal LC. While this material exhibited intermediate compressive strength (comparable to MTA and Biodentine), it showed significantly higher shear bond strength to composite resin than the hydraulic cements. This divergence can be explained by the fundamental difference between these mechanical properties. Compressive strength primarily reflects the material's internal cohesion and structural integrity, whereas shear bond strength depends on adhesive interfacial properties between dissimilar materials.

For TheraCal LC, this discrepancy suggests that while its resin matrix provides sufficient cohesion for adequate compressive strength, its chemical affinity with resin-based composites enables superior bonding performance. The material's resinous composition facilitates co-polymerization and mechanical interlocking with the adhesive system, creating a stronger interface than what can be achieved with the water-based cements despite surface etching (23, 24). This explains why TheraCal LC performs similarly to hydraulic cements in compression but excels in bond strength applications.

TheraCal PT outperformed all other materials in both mechanical tests, likely due to its dual-cure system that combines the benefits of chemical and light-activated polymerization, resulting in more complete conversion and enhanced mechanical properties throughout the material bulk (17, 20).

Regarding bioactive properties, MTA and Biodentine demonstrated superior biomineralization capacity with abundant calcium phosphate crystal formation on their surfaces. This aligns with their known mechanisms of action, involving continuous release of calcium and hydroxide ions that create an environment conducive to hydroxyapatite nucleation (25, 26). In contrast, TheraCal LC and PT showed minimal surface mineralization, suggesting their resin matrices may impede ion release and crystal growth, potentially limiting their long-term bioactive potential (27).

The cytocompatibility assessment revealed another critical trade-off. MTA exhibited excellent biocompatibility with hDPSCs, consistent with its well-established bioactivity and tissue-friendly properties (28, 29). Biodentine showed reduced viability, possibly due to calcium chloride in its accelerator liquid (30). Most notably, both resin-based materials demonstrated significant cytotoxicity, likely attributable to leachable monomers and photoinitiators that can disrupt cellular metabolism (19). This finding underscores the biological limitations of resin-modified materials despite their mechanical advantages.

Clinical implications of these results suggest that material selection should consider the specific clinical scenario. TheraCal PT may be preferable in situations requiring high mechanical strength and bond strength, while MTA remains the gold standard for cases where bioactivity and biocompatibility are paramount. TheraCal LC presents a middle ground with good bonding capability but compromised bioactivity and cytotoxicity concerns.

## 5 Conclusions

In summary, this study elucidated key properties of pulp capping materials. While resin-based materials showed superior bond strength to composite, water-based bioceramics still achieved notable adhesion through surface treatment. Radiographically, MTA and TheraCal LC demonstrated adequate visibility. Crucially, MTA exhibited excellent biocompatibility and biomineralization potential, essential for pulp healing. In contrast, Biodentine displayed reduced cellular viability, and the resin-based materials presented inherent cytotoxic concerns, underscoring the critical balance between physical performance and biological safety in selecting optimal materials for vital pulp therapy.

## Data Availability

The original contributions presented in the study are included in the article/Supplementary Material, further inquiries can be directed to the corresponding author.
